# Age-related differences in the intake of heart failure therapy among participants from the MyoVasc study

**DOI:** 10.1007/s41999-026-01553-4

**Published:** 2026-07-14

**Authors:** Michael Mohr, Silav Zeid, Gregor Buch, Felix Rausch, David Velmeden, Fawad Kazemi-Asrar, Benedikt Fooß, Karl J. Lackner, Tommaso Gori, Philipp Lurz, Jürgen H. Prochaska, Roland Hardt, Philipp S. Wild

**Affiliations:** 1 Geriatric Department, University Medical Center of the Johannes Gutenberg University Mainz, Mainz, Germany; 2 Preventive Cardiology and Preventive Medicine, Department of Cardiology, University Medical Center of the Johannes Gutenberg University Mainz, Langenbeckstr. 1, 55131 Mainz, Germany; 3 German Center for Cardiovascular Research (DZHK), Partner Site Rhine-Main, Mainz, Germany; 4 Institute of Medical Biostatistics, Epidemiology and Informatics (IMBEI), University Medical Center of the Johannes Gutenberg University Mainz, Mainz, Germany; 5 Institute of Clinical Chemistry and Laboratory Medicine, University Medical Center of the Johannes Gutenberg University Mainz, Mainz, Germany; 6 Department of Cardiology, University Medical Center of the Johannes Gutenberg University Mainz, Mainz, Germany; 7 Clinical Epidemiology and Systems Medicine – Center for Thrombosis and Hemostasis (CTH), University Medical Center of the Johannes Gutenberg University Mainz, Mainz, Germany; 8 Boehringer Ingelheim, Ingelheim, Germany; 9 Institute of Molecular Biology (IMB), Mainz, Germany

**Keywords:** Heart failure, Geriatric, Guideline-based therapy

## Abstract

**Key summary points:**

**Aim:**

To investigate whether older individuals (≥70 years) with heart failure receive guideline-recommended heartfailure medications less frequently than younger individuals.

**Findings:**

Older individuals had a higher prevalence of heart failure and greater polypharmacy but were treated lessoften with guideline-based therapies, including aldosterone antagonists and beta-blockers, even after adjustment forrenal function and comorbidities. In contrast, calcium channel blockers were prescribed more frequently in geriatricpatients.

**Message:**

Older individuals with heart failure appear to be undertreated with evidence-based therapies, highlightingage-related gaps in guideline-concordant care.

**Background:**

In Germany, heart failure (HF) is the most common diagnosis in hospitalized patients. Its prevalence rises significantly with age. In addition, age-related conditions, such as hypertension, coronary artery disease, obesity, and diabetes mellitus, contribute to an elevated risk of heart failure. It is assumed that older patients are more likely to receive treatment that does not conform to the guidelines because of frequent comorbidities, such as chronic renal disease and pre-existing polypharmacy.

**Methods:**

Data from a prospective cohort study on chronic HF (MyoVasc study, *N* = 3289; NCT04064450), were analyzed. Participants underwent a 5-h highly standardized examination, including assessment of medication with subsequent categorization according to the anatomical therapeutic chemical (ATC) coding system. Study participants were categorized as older adults aged ≥ 70 years and subsequently compared to younger adults regarding frequency of medication intake. Poisson regression with robust standard errors with adjustment for age, sex, cardiovascular risk factors, comorbidities, and kidney function was used to analyze medication intake by age status in subjects with chronic HF stages C/D.

**Results:**

The analyzed sample comprised 1281 older adults (mean age: 75.4 ± 3.6 years, 33.1% women, 66.9% men) and 2,008 younger adults (mean age: 57.7 ± 8.5 years, 35,2% women, 64.8% men). A total of 68.5% (*n* = 878) of the older individuals presented with HF stage C/D, compared to 43% (*n* = 863) in younger adults. On average, older individuals took 6.53 (standard deviation (SD) ± 3.14) drugs as compared to younger participants taking 4.65 (SD ± 3.45) drugs regardless of the severity of HF. Poisson regression analysis demonstrated that older individuals received aldosterone antagonists less frequently (Prevalence Ratio (PR): 0.64, 95%CI 0.54; 0.77, *p* < 0.0001). This effect persisted after additional adjustment for kidney function (PR: 0.53, 95%CI 0.44; 0.63, *p* < 0.0001). Beta-blockers were used significantly less frequently in older adults compared to younger adults (PR: 0.93, 95%CI 0.88; 0.99, *p* = 0.016). In contrast, calcium channel blockers were prescribed significantly more often in the older group (PR: 1.22, 95%CI 1.01; 1.48, *p* = 0.035). Additionally, older adults with impaired renal function received loop diuretics less frequently (PR: 0.88, 95%CI 0.78; 0.99, *p* = 0.033).

**Conclusion:**

The present study identified age-related disparities in the use of guideline-based therapy for heart failure. These differences persisted even after adjusting for renal dysfunction, a common and relevant comorbidity in the aged population. These findings suggest a potential underutilization of evidence-based heart failure therapies in older individuals with heart failure.

**Trial registry:**

NCT04064450

**Supplementary Information:**

The online version contains supplementary material available at https://doi.org/10.1007/s41999-026-01553-4.

## Introduction

Heart failure (HF) is a syndrome that is gaining increasing importance worldwide. Its prevalence rises significantly with age. In addition, age-related conditions, such as hypertension, coronary artery disease, obesity, and diabetes mellitus, contribute to an elevated risk of heart failure. Studies indicate that the prevalence of heart failure escalates significantly with each decade of life [1]. In individuals aged from 40 to 59 years, the prevalence stands at about 2%, while in those over 80 years, it surges to over 15 percent [2, 3] .

**Fig. 1:**
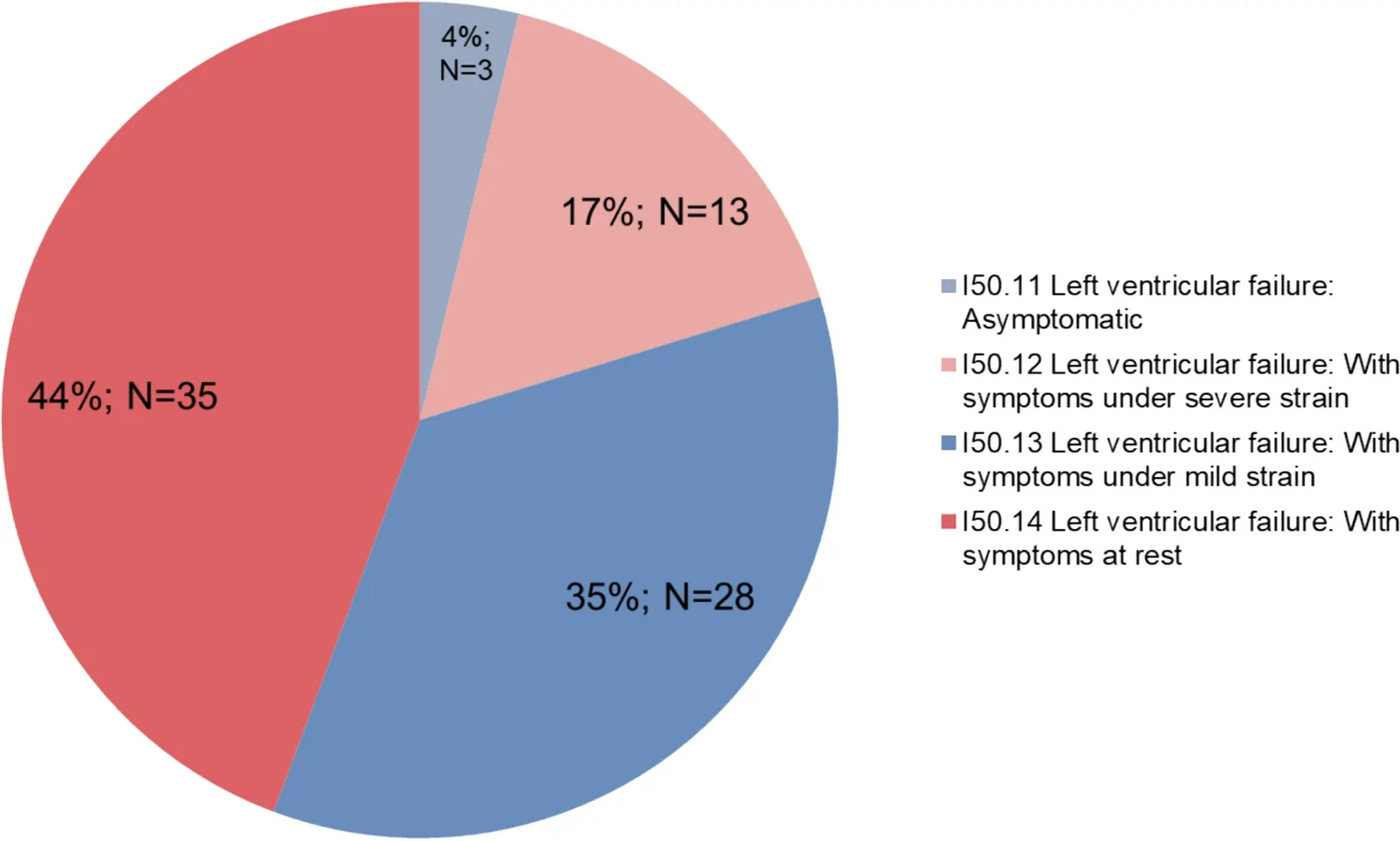
Pie chart with ICD-10 coded (I50.11–14) symptom burden in patients diagnosed with heart failure from the geriatric department at the University Medical Center Mainz, Germany. Abbreviations: ICD-10, International Classification of Diseases, Tenth Revision

In 2021, approximately 24% of inpatients in the geriatric department of the University Medical Center of the Johannes Gutenberg University Mainz (UMCM) were diagnosed with HF (I50, International Classification of Diseases, Tenth Revision [ICD-10]) regardless of their primary diagnosis. Among these patients, 52% exhibited symptoms during either mild (I50.12) or severe strain (I50.13) (Fig. 1). The treatment of HF in geriatric patients represents a challenge due to multi-morbidity, polypharmacy, and associated adverse drug reactions and side effects. [4] While older individuals with HFrEF benefit from guideline-based therapy, evidence suggests that such therapy is often underutilized in this patient population as age increases. [5] Among the “foundational four” drug classes reducing HF-related hospitalization or death, beta-blockers, mineralocorticoid receptor antagonists (MRAs), sacubitril–valsartan, dapagliflozin, and empagliflozin maintained their overall benefit–risk profile in terms of safety regardless of age. [6] Furthermore, there is evidence that older adults with HF, especially those classified as very old, may benefit more from guideline-based therapy to reduce the risk of mortality compared to no guideline-directed medical therapy. [7] Nevertheless, increasing age appears to be an important factor that leads to medications either not being prescribed or not being prescribed in adequate doses [8] .

Building on the challenges associated with treating HF in older patients, the impact of renal insufficiency on medication administration emerges as another crucial consideration in this particularly vulnerable population. Chronic kidney disease (CKD) becomes increasingly important in old age [9] and appears to be a reason for the restricted administration of some medications in older adults. Anjum et al. (2023) showed that HF patients with severe kidney dysfunction (eGFR < 30) had a significantly lower initiation rate of therapy with MRA, beta-blockers and renin-angiotensin system (RAS) inhibitors. The underuse of RAS inhibitors in moderate CKD in this study was mainly influenced by age and frailty [10]. Furthermore, older adults with heart failure are at significant risk of kidney complications, including the need for dialysis, if they have previously been hospitalized for heart failure (HF) [11] .

Expanding on the implications of CKD for medication administration in the aged HF population, this study aims to investigate whether older adults with heart failure are more likely to receive treatment that does not align with current therapy guideline recommendations due to prevalent multi-morbidity, such as chronic renal disease and pre-existing polypharmacy.

## Methods

### Study design

Baseline data from the MyoVasc study (*N* = 3 289), a prospective cohort study on individuals with HF (NCT04064450), were analyzed. Recruitment for the MyoVasc Study began in 2013 and was completed in 2018. Transthoracic echocardiography was conducted at the MyoVasc study center as part of the examination to evaluate cardiac status. A comprehensive overview of the design and rationale for the MyoVasc study, along with baseline characteristics, has been published elsewhere. [12] Before commencing this study, the study protocol was approved by the local data protection officer and the responsible ethics committee (*Ethik-Kommission der Landesärztekammer Rheinland-Pfalz*, reference number 837.319.12 [8420-F]). Prior to enrollment, all study participants provided written informed consent. The study procedures adhered to the Declaration of Helsinki [13] as well as the recommendations of good clinical and good epidemiological practice. Participants were recruited from hospitals, medical practices, and through random sampling from registry offices.

### Heart failure classification

Participants in the MyoVasc study were classified according to current heart failure guidelines and the Universal Definition of Heart Failure [14–16], including individuals without evidence of cardiovascular disease (controls), individuals at risk for heart failure (Stage A), individuals with pre-heart failure characterized by structural heart disease but no symptoms (Stage B), and patients with established heart failure (Stage C or D). Classification was based on an integrated clinical assessment, including medical history and documentation from treating physicians, evaluation of typical signs and symptoms of heart failure, and objective assessment of cardiac structure and function by transthoracic echocardiography performed at the study center. Echocardiography was used to further phenotype heart failure into heart failure with reduced (HFrEF), mildly reduced (HFmrEF), and preserved ejection fraction (HFpEF). Functional status was assessed using the New York Heart Association (NYHA) classification. Natriuretic peptide measurements along with other relevant cardiac blood-based biomarkers were assessed as part of the clinical evaluation during the study visit.

### Participant examination

Participants underwent a highly standardized 5-h examination at a dedicated study center, which included the assessment of cardiac structure and function by echocardiography, along with anthropometric measurements, demographic data collection, and medical history assessment. Medication intake was categorized based on the Anatomical Therapeutic Chemical Classification System (ATC). Venous blood was drawn to measure routine blood markers relevant to traditional cardiovascular risk factors (CVRFs) and the HF syndrome in particular. The presence of CVRFs (i.e., arterial hypertension, diabetes mellitus, smoking, obesity, dyslipidemia, and family history of ischemic stroke or myocardial infarction [MI]) and comorbidities (including MI, stroke, coronary artery disease [CAD], peripheral artery disease [PAD] defined by physicians’ diagnosis or ankle–brachial index [ABI] < 0.9, cancer, chronic kidney disease defined by physicians’ diagnosis or estimated glomerular filtration rate [eGFR] < 60 ml/min/1.73 m2 [CKD], venous thromboembolism [VTE], and chronic obstructive pulmonary disease [COPD]) was assessed through a thorough computer-assisted personal interview and confirmed by laboratory markers and clinical investigations where applicable.

### Statistical analysis

The analyzed sample included individuals at risk of HF (HF stages 0 or A), pre-heart failure (HF stage B), and symptomatic HF (HF stage C or D). Study participants were categorized as older adults when aged ≥ 70 years and younger when aged < 70 years. Baseline characteristics of the individuals with HF were stratified by being older or younger. The age of the study participants was reported as mean [standard deviation, (SD)], and discrete variables were described by relative and absolute frequencies.

The distribution of the number of concomitant medication intake was stratified by age for both the analysis sample as well as the subsample with symptomatic HF only. Next, to show the difference in cardiovascular drug intake between older and younger participants, Poisson regression with robust standard errors with adjustment for age, sex, CVRFs and comorbidities was used to analyze the medication intake by age status in the subsample with HF, and the HF phenotypes, i.e., HF with preserved ejection fraction [EF] (HFpEF), HF with mildly reduced EF (HFmrEF), and HF with reduced EF (HFrEF). In a second model, there was an additional adjustment for the estimated glomerular filtration rate (eGFR) to account for renal function. This analysis was repeated in sex-stratified subgroups (women and men). Sensitivity analysis was performed in participants aged 80 or older versus younger participants.

## Results

### Baseline characteristics

A total of 1,741 individuals with HF were included for analysis, of whom 878 were older (mean age: 75.7 ± 3.7 years) and 863 were younger patients (mean age: 59.7 ± 7.4 years). Older participants showed a higher prevalence of CVRFs than younger (Table 1). Only the proportion of active smokers and obesity was lower in older vs. younger participants. Similarly, the prevalence of chronic and cardiovascular disease was higher in older vs. younger participants (Table 1). In terms of functional classification, older patients exhibited a higher prevalence of limitations in physical activity (21.2%) compared to younger patients (17.1%). Furthermore, within the HF phenotypes, the older patients group showed a higher prevalence of individuals with HFpEF (56.3%) compared to the younger group (41.8%). Supplementary Table 1 summarizes baseline medication use as number and proportion of participants receiving each ATC-class medication, stratified by age group. Compared with younger participants, older individuals with HF more frequently received nitrates, diuretics, loop diuretics, and calcium channel blockers, whereas aldosterone antagonists and ACE inhibitors were prescribed less frequently. In contrast, use of beta-blockers, agents acting on the renin–angiotensin system overall, antiarrhythmic agents, and cardiac glycosides, was broadly comparable between age groups.

**Table 1:** Baseline characteristics stratified by geriatric vs. non-geriatric individuals with heart failure

		Individuals aged < 70 (*n* = 863)		Individuals aged ≥ 70 years (*n* = 878)	*P*
Demographics and BMI					
Sex, women [%] (*n*)		33.1 (286)		35.2 (309)	0.39
Age [years], mean ± SD		59.7 ± 7.4		75.7 ± 3.7	< 0.0001
Body mass index [kg/m^2^]		29.8 ± 5.6		28.5 ± 4.6	< 0.0001
Cardiovascular risk factors [%] (*n*)					
Active smoking		13.2 (185)		3.6 (36)	< 0.0001
Arterial hypertension		61.7 (869)		83.5 (847)	< 0.0001
Diabetes mellitus		16.8 (233)		26.7 (268)	< 0.0001
Dyslipidemia		47.3 (659)		63.6 (640)	< 0.0001
Family history of MI or stroke		24.6 (346)		33.6 (341)	< 0.0001
Obesity		31.0 (434)		28.2 (285)	0.14
Clinical profile [%] (*n*)					
Atrial fibrillation		16.0 (224)		33.8 (340)	< 0.0001
Coronary artery disease [%] (*n*)		28.7(224)		45.1 (453)	< 0.0001
History of coronary artery bypass grafting		8.5 (119)		15.4 (156)	< 0.0001
History of myocardial infarction		20.3 (285)		25.7 (259)	0.0019
History of PCI		26.1 (367)		39.5 (401)	< 0.0001
Chronic kidney disease		14.0 (196)		22.5 (225)	< 0.0001
Chronic obstructive pulmonary disease		15.4 (216)		15.1 (152)	0.86
History of cancer		12.1 (171)		25.9 (261)	< 0.0001
History of stroke		5.7 (80)		10.7 (108)	< 0.0001
History of transient ischemic attack		4.0 (57)		7.9 (80)	< 0.0001
History of venous thromboembolism		6.2 (87)		10.3 (104)	0.00031
ICD or cardiac resynchronization therapy		7.7 (109)		7.3 (74)	0.76
Pacemaker		4.5 (63)		11.0 (111)	< 0.0001
Peripheral artery disease [%] (*n*)		4.9 (68)		8.2 (81)	0.0014
Heart failure status					
E/E ‘-ratio, median (IQR)		9.00 (6.74/12.25)		10.71 (8.10/14.05)	< 0.0001
LVEF [%], mean ± SD		48.7 ± 12.2		51.3 ± 11.3	< 0.0001
NT-proBNP [pg/ml], median(IQR)		265.0 (134.0/735.6)		508.0 (222.7/1232.7)	< 0.0001
eGFR [ml/min/1.73m^2^], mean ± SD		79.14 ± 20.26		64.30 ± 18.40	< 0.0001
Heart failure phenotype [%] (*n*)					
HfpEF		41.8 (295)		56.3 (431)	
HfmrEF		31.1 (219)		23.8 (182)	
HfrEF		27.1 (191)		20.0 (153)	
NYHA functional class [%] (*n*)					
NYHA I		45.7 (394)		43.3 (380)	
NYHA II		37.2 (321)		35.5 (312)	
NYHA III + IV		17.1 (148)		21.2 (186)	

MI, myocardial infarction; PCI, percutaneous coronary intervention; ICD, implantable cardioverter-defibrillator; E/E’-ratio, the ratio of early diastolic mitral inflow velocity to early diastolic mitral annulus velocity; *LVEF* left ventricular ejection fraction, *eGFR* estimated glomerular filtration rate, *NYHA* New York Heart Association functional classification

### Medication

**Fig. 2:**
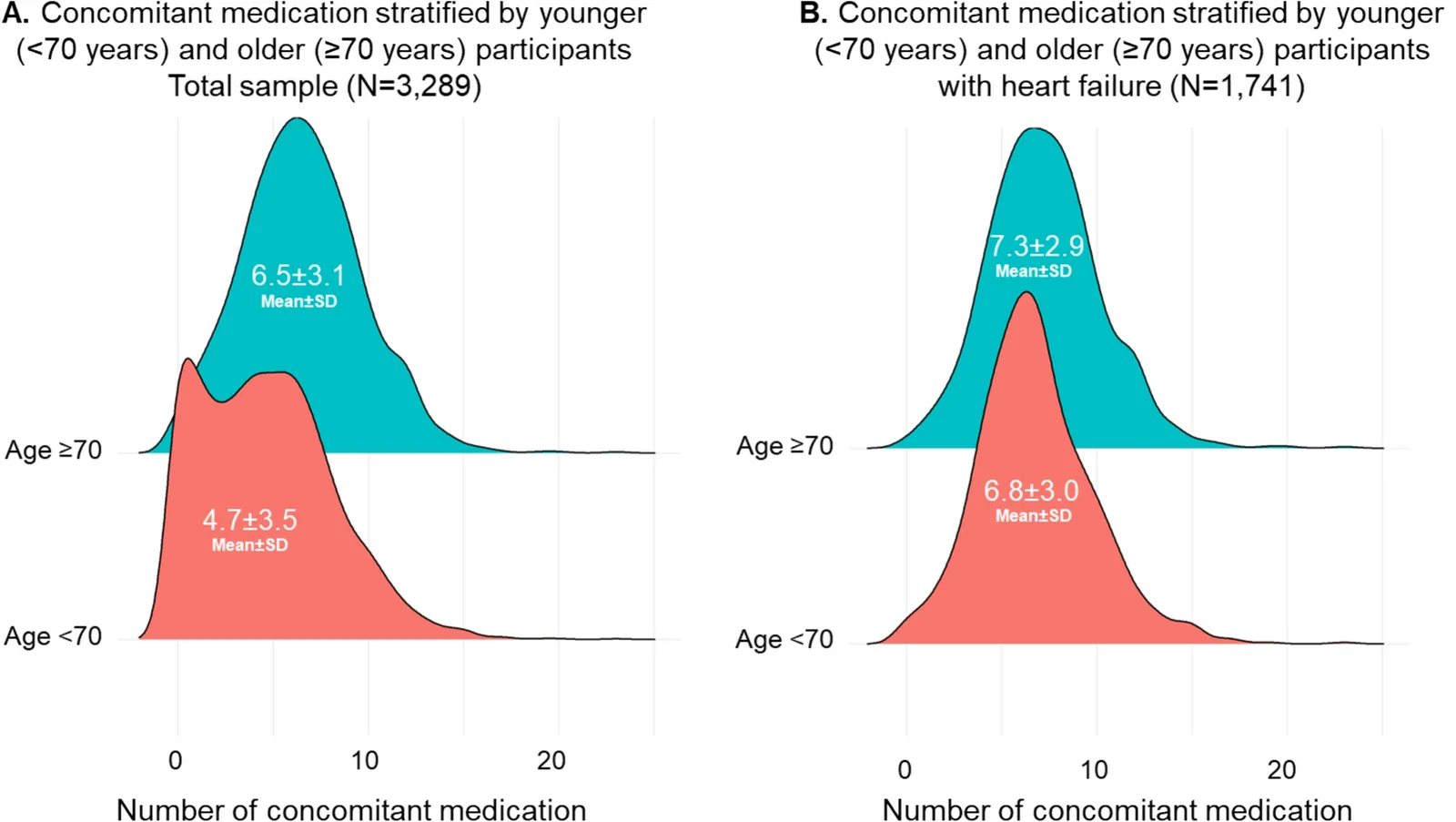
Distribution of the number of concomitant medication intake stratified by age (in blue: ≥ 70 years vs in orange: < 70 years). Panel A: Distribution of the number of concomitant medication intake in the analysis sample. Panel B: Distribution of the number of concomitant medication intake in the subsample with symptomatic heart failure (stage C/D)

**Fig. 3:**
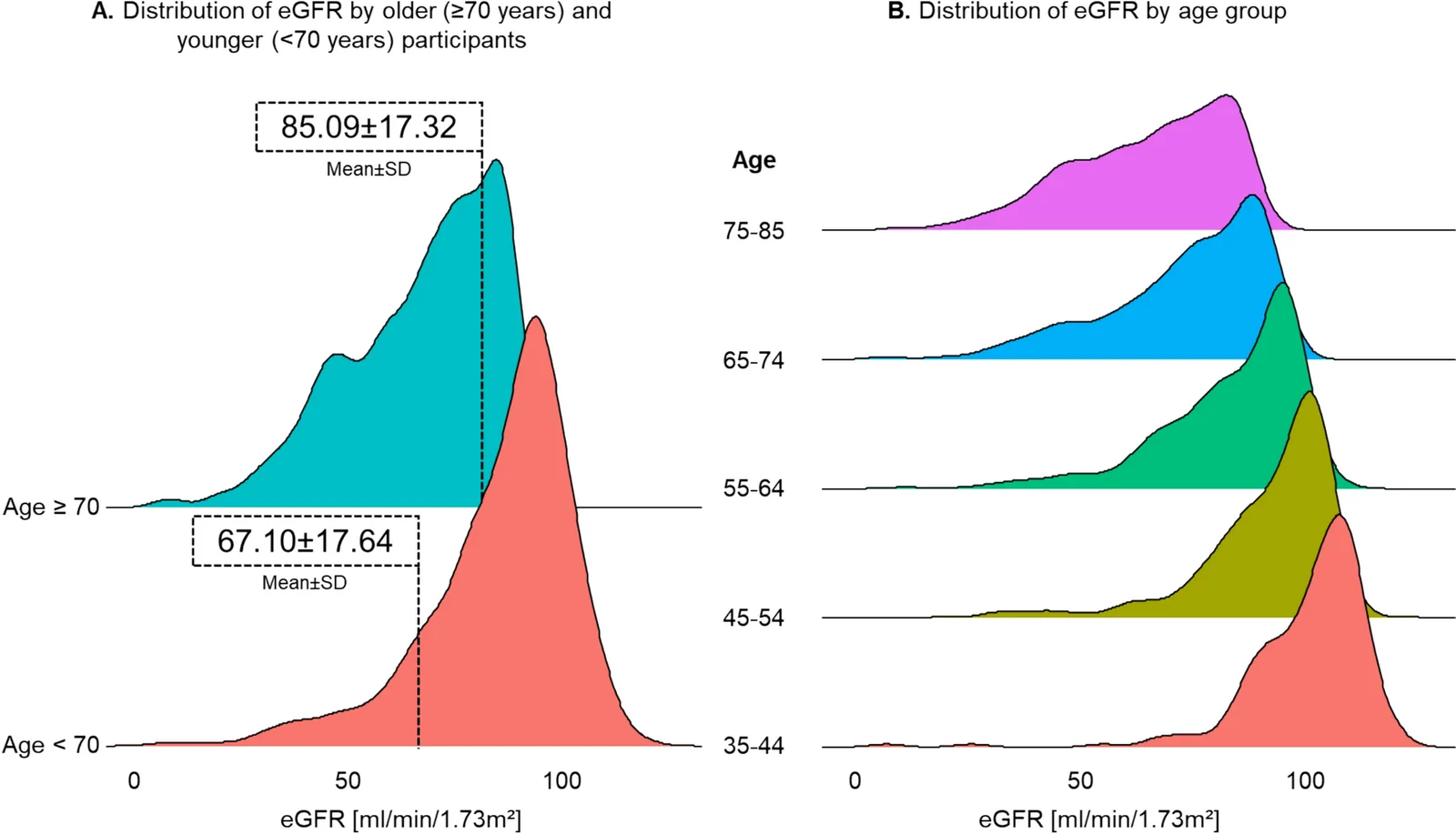
Distribution of eGFR by old vs younger participants and heart failure stage

Participants aged ≥ 70 years took on average approximately 6.5 (± 3.14) medications, whereas this number was 4.7 (± 3.45) for those < 70 years old (Fig. 2, Panel A). The number of concomitant medications in individuals with HF was higher than in the analysis sample regardless of age (Fig. 2, Panel B). Renal function was worse in older participants compared to younger participants (Fig. 2, Panel A). Within older participants, eGFR decreased with age (Fig. 3, Panel B).

**Fig. 4:**
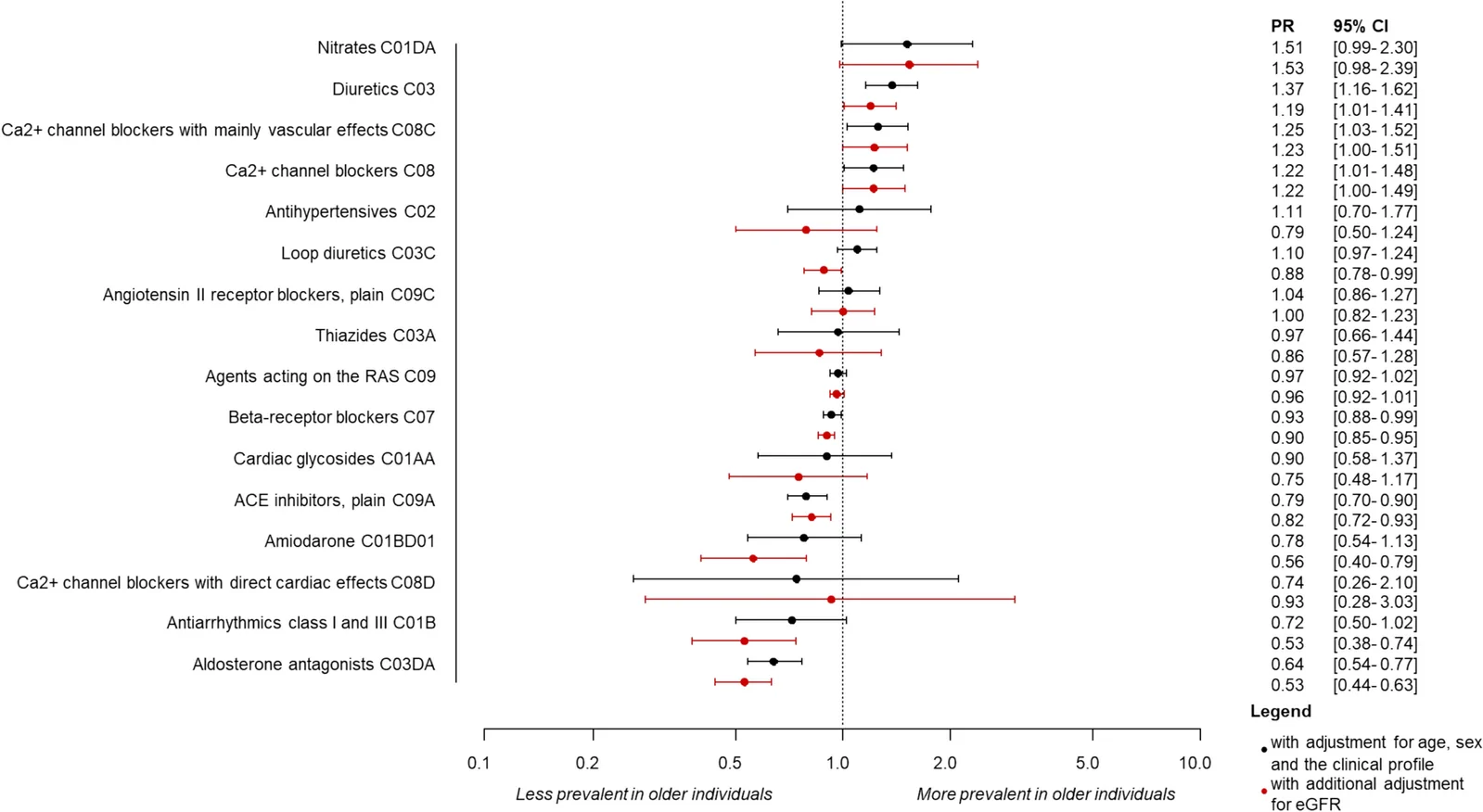
Difference in cardiovascular drug intake between older and youngerc patients in the HF subsample. Results from Poisson regression models with adjustment for age, sex, and the clinical profile (i.e., arterial hypertension, diabetes mellitus, obesity, dyslipidemia, history of myocardial infarction/stroke, history of smoking, myocardial infarction, stroke, atrial fibrillation, chronic obstructive pulmonary disease, coronary artery disease, peripheral artery disease, venous thromboembolism, and chronic kidney disease) in black, and with additional adjustment for eGFR in red

Figure 4 shows the difference in cardiovascular drug intake between older and younger individuals with HF. Older individuals with HF took more diuretics (Prevalence Ratio [PR]: 1.37, confidence interval 95% [CI] 1.16; 1.62). Conversely, older individuals with heart failure received fewer β-receptor blockers (PR: 0.93, CI: 0.88; 0.99) and aldosterone antagonists (PR: 0.64, CI: 0.50–0.77). There was also a non-significant difference regarding intake of loop diuretics (PR: 1.10, CI: 0.97; 1.24), nitrates, and calcium channel blockers (Fig. 4). After additional adjustments for eGFR, a significantly lower use of beta-blockers and aldosterone antagonists were observed in older individuals with heart failure (Fig. 4). For aldosterone antagonists, the effect was amplified (PR: 0.53, CI 0.44; 0.63). With regard to loop diuretics, there was a reversal effect after adjustments for eGFR (PR 0.88 CI 0.78–0.99). Supplementary Fig. 1 shows the sensitivity analysis in individuals aged older than 80 years old versus younger individuals. In this analysis, prevalence ratios are similar but attenuated. Sex-stratified analysis demonstrated broadly consistent findings in men and women, without evidence of major qualitative differences. Confidence intervals were wider in women, most likely due to a smaller subgroup size. Results are shown in Supplementary Fig. 2 (men) and Supplementary Fig. 3 (women).

**Fig. 5:**
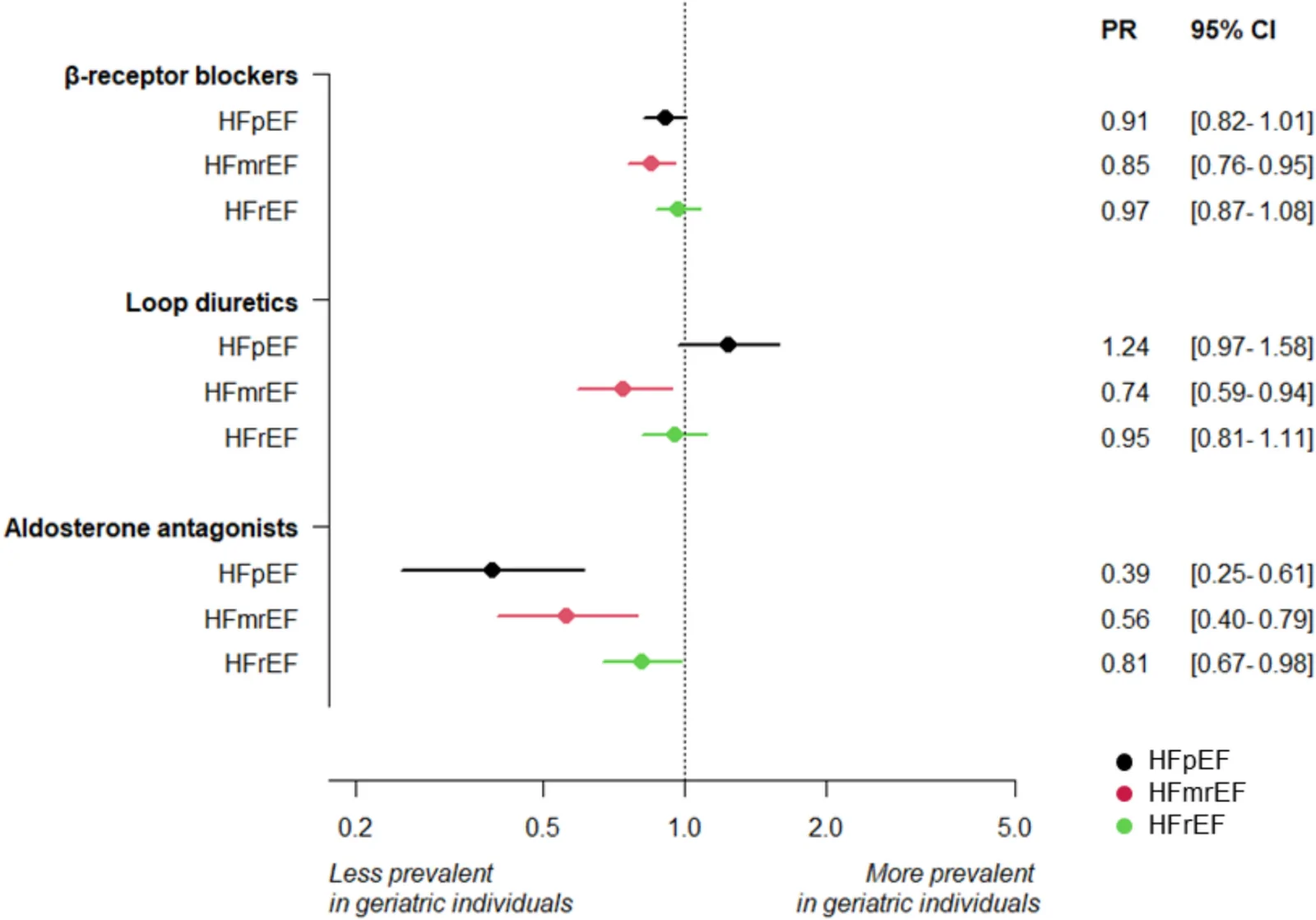
Difference in drug intake between older and younger patients according to the heart failure phenotypes. Results from Poisson regression models with adjustment for age, sex, the clinical profile (i.e., arterial hypertension, diabetes mellitus, obesity, dyslipidemia, history of myocardial infarction/stroke, history of smoking, myocardial infarction, stroke, atrial fibrillation, chronic obstructive pulmonary disease, coronary artery disease, peripheral artery disease, venous thromboembolism, and chronic kidney disease) and eGFR. In black, the subsample with heart failure (HF) with preserved ejection fraction (EF; HFpEF); in red, the subsample with HF with mildly reduced EF (HFmrEF); and in green, the subsample with HF with reduced EF (HFrEF)

Next, analyses were repeated within the HF phenotypes. Evaluation confirmed the significantly lower use of aldosterone antagonists across all phenotypes, particularly in individuals with HFpEF (Fig. 5). Beta-blocker use was reduced across all HF phenotypes. However, this difference was statistically significant only in participants with HFmrEF. Individuals with HFpEF, compared to individuals with HFmrEF and HFrEF, took loop diuretics more frequently (Fig. 5). Supplementary Fig. 4 shows the corresponding analyses at the ATC subclass level, offering a more detailed classification of medication use. These findings were consistent with the results from the aggregated ATC class-level analysis.

## Discussion

It is well established that the prevalence of heart failure (HF) increases with age, and that there are differences in the phenotype of HF. In the older group, the proportion of individuals with HFpEF was significantly higher than in younger individuals, which is consistent with the findings of previous studies. [17, 18] HFpEF is the leading form of HF in older people. [19] The prevalence of HFpEF in individuals aged 80 years and older exceeds 10% [17, 20] .

The analysis of data from the MyoVasc study regarding the treatment of older individuals with HF contributed to a better understanding of drug therapy in geriatric individuals with symptomatic HF. This indicates a possible undersupply of HF medications in older patients. Furthermore, a body of evidence exists indicating that geriatric patients, particularly those classified as aged 80 years and above, frequently do not undergo treatment for heart failure in accordance with established clinical guidelines. [21] It is frequently overlooked that older patients may also derive comparable benefits from guideline-based therapy for HF [6] .

In our study, aldosterone antagonists (MRA), beta-blockers, and loop diuretics were used to a substantially lesser extent in older participants with HF. In the case of MRA, this association was independent of baseline renal function and HF phenotype, which is surprising, since older people take a higher number of medications overall (polypharmacy). The subgroup analyses according to HF phenotype, sex, and age stratification at 80 years were limited by the smaller sample sizes in these groups. In this context, it should be mentioned that the older participants with HF were relatively young (mean: 75.7 ± 3.7 years). In clinical practice, older patients are usually characterized by an age > 80 years [22]. In this context, it should be noted that a comprehensive geriatric assessment (CGA), an important aspect of geriatric care, was not performed on the study population. Consequently, no conclusions could be drawn regarding frailty, which is a treatable and potentially reversible syndrome that is prevalent among older patients with HF and increases the risk of disability and other adverse health outcomes [23] .

Renal function was considered a key element in the treatment decision in this study. In particular, there appears to be a concern regarding the use of MRA in older individuals with reduced renal function as there is a fear that this could lead to a further worsening in renal function (WRF). This effect has been confirmed by previous studies. [24] Although adverse effects such as WRF or hyperkalemia are more frequently reported in older patients, they still benefit from MRA therapy to a similar extent as younger patients with HF, irrespective of the HF phenotype. [25] Notably, it has been shown that this approach results in a reduced risk of all-cause mortality even when accompanied by WRF [26]. These effects can be observed irrespective of the parameter utilized for renal function assessment (eGFR or creatinine). [27] For individuals with mildly reduced or preserved ejection fraction, it has been shown that the clinical benefits of finerenone (MRA) outweigh the risks, even when potassium levels rise during treatment. [28] Patients with HFpEF have also been shown to have a reduction in cardiovascular death despite WRF [29]. Holmdahl et al. (2021) demonstrated that the use of MRA in older individuals with HFrEF does not elevate the risk of renal insufficiency deterioration and that there was no increased mortality observed. [30] A potential decline in renal function during the course of therapy with an MRA should not be a rationale for the withholding of this therapy from geriatric patients. Instead, renal function and potassium levels should be monitored meticulously throughout the course of therapy. The problem of hyperkalemia during MRA therapy can be addressed by the use of potassium binders such as patiromer [31] .

In regard to the use of loop diuretics, it was observed that individuals with HFpEF were more likely to be prescribed these medications, while the opposite was true for individuals with HFrEF and HFmrEF. In the case of HFpEF, it was demonstrated that age was one of the most significant factors associated with the use of loop diuretics [32] .

Regardless of HF therapy, it was seen that use of nitrates and calcium channel blockers was higher in older patients. Due to the long medical history of geriatric patients, medication may not have been adjusted to current guidelines, which explains the increased use of these preparations in this patient population.

Future studies with larger cohorts could help determine whether similar effects are observed with ARNI and SGLT-2 inhibitors. SGLT-2 inhibitors, in particular, have become a critical component of the therapeutic approach for HFrEF and HFpEF, irrespective of the presence of diabetes mellitus. [33]. This treatment has also been shown to be advantageous for older patients with multiple chronic conditions, with a notable reduction in the risk of heart failure rehospitalization or cardiovascular death. [34] Furthermore, recent studies have demonstrated that ARNI is now a viable treatment option for all phenotypes of heart failure although patients with HFrEF in particular benefit from it. [35] It appears that gender is a factor in the utilization of this class of drugs [36] .

This study highlights significant differences in the pharmacological treatment of HF between older and younger patients, emphasizing potential underuse of guideline-based medical therapy in older adults. Despite adjustment for renal function and other relevant factors, older patients were less likely to receive aldosterone antagonists and beta-blockers, while calcium channel blockers were more frequently prescribed. These findings point to a need for increased awareness and efforts to optimize HF treatment in older patients, taking into account their comorbidities and polypharmacy. Addressing these gaps may help improve clinical outcomes in this vulnerable population.

## Limitations

This study is limited by its small sample size of individuals aged over 70 years old, particularly in subgroup analysis by HF phenotype, and in the very old (> 80) population with heart failure. In addition, the number of very old individuals was limited, and the cohort was comparatively young (mean age 75.7 ± 3.7 years), potentially leading to an underestimation of treatment differences in more advanced age groups. Furthermore, no comprehensive geriatric assessment was performed, precluding adjustment for frailty, functional status, and cognitive impairment. Finally, as the cohort was recruited between 2013 and 2018, newer guideline-directed data on newer HF therapies, such as angiotensin receptor–neprilysin inhibitors and SGLT-2 inhibitors, were not routinely available, limiting conclusions regarding restricting conclusions on contemporary treatment patterns.

## Supplementary Information

Below is the link to the electronic supplementary material.

Supplementary file1 (DOCX 247 KB)

## Data Availability

This project constitutes a major scientific effort with high methodological standards and detailed guidelines for analysis and publication. Data are not made available for the scientific community outside the established and controlled workflows and algorithms. To meet the general idea of verification and reproducibility of scientific findings, we offer access to data at the local database in accordance with the ethics vote on request (contact: Prof. Dr. Philipp Wild (PI), philipp.wild@unimedizin-mainz.de).
