# Supplementary material for: Age-related differences in the intake of heart failure therapy among participants from the MyoVasc study
Source: Eur Geriatr Med. 2026 Jul 14;17(4):2087–97. doi: 10.1007/s41999-026-01553-4 (PMC13562216; doi:10.1007/s41999-026-01553-4)
Supplement: Supplementary file 1 [file 41999_2026_1553_MOESM1_ESM.docx]

**Age-related differences in the intake of heart failure therapy among participants from the MyoVasc study**

Michael Mohr^1^, Silav Zeid^2,3^, Gregor Buch^2,3,4^, Felix Rausch^2,3,6,7^, David Velmeden^2,3^, Fawad Kazemi-Asrar^2,3^, Benedikt Fooß^2,3^, Karl J. Lackner^5,3^, Tommaso Gori^6,3^, Philipp Lurz^6,3^, Jürgen H. Prochaska^2,3,7,8^, Roland Hardt^1^, Philipp S. Wild^2,3,7,8,9^

^1^ Geriatric Department, University Medical Center of the Johannes Gutenberg University Mainz, Mainz, Germany

^2^ Preventive Cardiology and Preventive Medicine, Department of Cardiology, University Medical Center of the Johannes Gutenberg University Mainz, Mainz, Germany

^3^ German Center for Cardiovascular Research (DZHK), Partner Site Rhine-Main, Mainz, Germany;

^4^ Institute of Medical Biostatistics, Epidemiology and Informatics (IMBEI), University Medical Center of the Johannes Gutenberg University Mainz, Mainz, Germany

^5^ Institute of Clinical Chemistry and Laboratory Medicine, University Medical Center of the Johannes Gutenberg University Mainz, Mainz, Germany

^6^ Department of Cardiology – Cardiology I, University Medical Center of the Johannes Gutenberg University Mainz, Mainz, Germany

^7^ Clinical Epidemiology and Systems Medicine – Center for Thrombosis and Hemostasis (CTH), University Medical Center of the Johannes Gutenberg University Mainz, Mainz, Germany;

^8^ Boehringer Ingelheim, Ingelheim, Germany;

^9^ Institute of Molecular Biology (IMB), Mainz, Germany.

**Address for correspondence**

Philipp Sebastian Wild, MD, MSc

Professor of Clinical Epidemiology

Preventive Cardiology and Preventive Medicine, Department of Cardiology and

Clinical Epidemiology and Systems Medicine, Center for Thrombosis and Hemostasis

University Medical Center of the Johannes Gutenberg-University Mainz

Langenbeckstr. 1, 55131 Mainz, Germany

Phone: +49 6131 17 7163

Email: philipp.wild@unimedizin-mainz.de

[**Supplementary Figure 1.** Difference in cardiovascular drug intake between younger <80 and older aged ≥80 individuals	3](#_Toc231401990)

[**Supplementary Figure 2.** Difference in cardiovascular drug intake between younger <70 and older aged ≥70 men	4](#_Toc231401991)

[**Supplementary Figure 3.** Difference in cardiovascular drug intake between younger <70 and older aged ≥70 women	5](#_Toc231401992)

[**Supplementary Figure 4.** Difference in intake of HF medication between younger <70 and older aged ≥70 individuals according to HF phenotype	6](#_Toc231401993)

[**Supplementary Table 1.** Baseline characteristics of medication intake in individuals with heart failure stratified by younger (<70 years) and older (≥70 years) age	7](#_Toc231401994)

# **Supplementary Figure 1.** Difference in cardiovascular drug intake between younger <80 and older aged ≥80 individuals

# **Supplementary Figure 2.** Difference in cardiovascular drug intake between younger <70 and older aged ≥70 men

# **Supplementary Figure 3.** Difference in cardiovascular drug intake between younger <70 and older aged ≥70 women

# **Supplementary Figure 4.** Difference in intake of HF medication between younger <70 and older aged ≥70 individuals according to HF phenotype

# **Supplementary Table 1.** Baseline characteristics of medication intake in individuals with heart failure stratified by younger (<70 years) and older (≥70 years) age

|  | Individuals with heart failure aged <70 years (n=863) | Individuals with heart failure aged ≥70 years (n=878) | **P value** |
| --- | --- | --- | --- |
| Cardiac glycosides, C01AA | 3.9% (34) | 5.0% (44) | 0.30 |
| Class I and III antiarrhythmic agents, C01B | 7.9% (68) | 7.1% (62) | 0.52 |
| Amiodarone, C01BD01 | 7.0% (60) | 6.7% (59) | 0.85 |
| Nitrates, C01DA | 4.3% (37) | 6.8% (60) | **0.022** |
| Antihypertensive agents, C02 | 3.7% (32) | 4.3% (38) | 0.54 |
| Diuretics, C03* | 20.3% (175) | 32.0% (281) | **<0.0001** |
| Loop diuretics, C03C | 33.7% (291) | 40.4% (355) | **0.0040** |
| Thiazide diuretics, C03A | 6.3% (54) | 6.3% (55) | 1.00 |
| Aldosterone antagonists, C03DA | 29.3% (253) | 18.5% (162) | **<0.0001** |
| Beta-blockers, C07 | 76.0% (656) | 74.1% (651) | 0.38 |
| Calcium channel blockers, C08 | 18.4% (159) | 24.5% (215) | **0.0024** |
| Vascular calcium channel blockers, C08C | 17.3% (149) | 23.3% (205) | **0.0019** |
| Cardiac calcium channel blockers, C08D | 1.0% (9) | 1.1% (10) | 1.00 |
| Agents acting on the RAS, C09 | 81.5% (703) | 80.5% (707) | 0.63 |
| ACE inhibitors, C09A | 43.9% (379) | 35.1% (308) | **0.00019** |
| Angiotensin II receptor blockers, C09C | 19.8% (171) | 22.2% (195) | 0.24 |

ATC codes provided with the drug class name. Abbreviations, RAS, renin–angiotensin system. *Diuretics (C03) excluding the category aldosterone antagonists (C03DA).
